# A prospective experimental study of liver fibrosis with ultrasound and its correlation with hepatic reserve function and hemodynamics

**DOI:** 10.1186/1471-230X-12-168

**Published:** 2012-11-23

**Authors:** Yi-Lin Yang, Li Di, Yun-You Duan, Xi Liu, Jie Liu, Rui-Jing Yang, Sheng Chen, Li-Jun Yuan

**Affiliations:** 1 Department of Ultrasound Diagnosis, Tangdu Hospital, Fourth Military Medical University, Xi’an, Shaanxi Province, 710038, China; 2 Department of Obstetrics and Gynaecology, Tangdu Hospital, Fourth Military Medical University, Xi’an, Shaanxi Province, 710038, China

**Keywords:** Hepatic circulation index, Liver fibrosis, Hepatic function reserve, Doppler

## Abstract

**Background:**

Progressive hepatic fibrosis is the eventual cause of liver cirrhosis. Doppler ultrasound has been used to detect hemodynamic changes that are known to be present during the pre-cirrhotic stages of hepatic fibrogenesis. However, the relationship between the Doppler ultrasound parameters and the impairment of the liver function has not been fully investigated. The purpose of this study was to explore the hepatic function reserve and its relationship with the hepatic hemodynamics in a rabbit model of liver fibrosis using Doppler ultrasound.

**Methods:**

A prospective study was performed. Sixty healthy New Zealand rabbits were included in this study. Eleven of them served as controls and were normally fed and provided with water drink; the rest of 49 rabbits that served as fibrosis group were normally fed but provided with 1.2 g/L of thioacetamide to create liver fibrosis model. Doppler measurements were performed in the portal trunk, proper hepatic artery and proper splenic artery. The hepatic circulation index (HCI) was calculated. Hepatic function reverse was evaluated by measuring the indocyanine green clearance and retention rate at 15 min (ICG R15) test. Portal venous pressure (PVP) was measured using the portal vein punctuation equipment.

**Results:**

HCI was significantly decreased and PVP increased in the advanced fibrotic stage (F4) compared to mild and moderate fibrotic stage (F1-3), respectively (p<0.05). PVP and ICG R15 in the fibrotic group were significantly higher than that in the control group (ICG: 0.209±0.086 vs. 0.093±0.023, p<0.01). Within the fibrotic groups, PVP was higher in advanced fibrotic stage (F4) than those in mild (F1-2) or moderate (F3) fibrotic stages (p<0.05). Both HCI and PVP correlated well with ICG R15 (*r* = −0.890, and *r* = 0.780, p <0.01).

**Conclusions:**

Hepatic function reserve closely relates to the hepatic hemodynamics in the rabbit model of liver fibrosis. Doppler Ultrasound could be reliably used to assess the hepatic function reserve and hemodynamic changes in different stages of liver fibrosis.

## Background

Progressive hepatic fibrosis is the eventual cause of liver cirrhosis. There is increasing evidence that, unlike cirrhosis, fibrosis is treatable and reversible in its early stages. Prompt and effective treatment could postpone or interrupt the development of chronic hepatitis into liver cirrhosis [1-4]. Thus assessment of hepatic functional reserve in patients with fibrosis is critical for predicting its prognosis and preventing postoperative liver failure.

Quantitative tests of hepatic function are thought to assess the functional hepatic mass by measuring the blood flow-dependent hepatocyte function, such as indocyanine green (ICG) clearance. It is considered to reflect the degree of sinusoidal capillarization, intrahepatic portovenous shunt and alteration in liver blood flow [2,3]. Earlier studies indicated that ICG clearance was a good marker of hepatocellular uptake function [4]. Altered ICG clearance is an extremely sensitive early indicator of hepatocyte abnormality [5]. Thus ICG clearance and retention rate at 15 min (ICG R15) test have been widely used to assess liver function reserve in patients with chronic liver diseases, and to evaluate the liver function in organ donors and recipients and in critically ill patients.

At present, the diagnosis of liver fibrosis still depends on pathological examination of the liver tissue. Since this method is invasive, its application and extensive use in clinical practice are limited to diagnose and stage liver diseases affecting large segments of the population or to monitor disease progression or treatment effects [6]. Thus, greater attention has been paid to search for non-invasive diagnostic parameters for assessing liver fibrosis.

Ultrasonography (US) is the first choice of imaging modality used in the clinic in patients with diffuse liver disease [7]. The use of gray and color Doppler US in diagnosis and staging of chronic liver disease is based on the hypothesis that alteration of liver parenchyma and hemodynamics may reflect indirectly the histological alterations. The combination of gray scale US and Doppler US improve the diagnostic accuracy and are essential for the diagnosis of cirrhosis or fibrosis [8,9]. Doppler US has been used to detect hemodynamic changes that are known to be present during the pre-cirrhotic stages of hepatic fibrogenesis. However, though US Doppler parameters in patients with hepatic fibrosis show differences when compared to controls, the relationship between these parameters and the impairment of the liver function has not been fully investigated. In the present study, we adopted an experimental New Zealand rabbit model of liver fibrosis to observe the hemodynamic changes at different stages of fibrosis by US, and to evaluate whether these Doppler parameters could reflect the liver functional reserve changes.

## Methods

### Animals

All experimental animals were purchased from the Animal Experimentation Center of Academy of Traditional Chinese Medicine, Shaan’xi, China. Sixty healthy New Zealand rabbits, weighed 2.5~3.0 kg, were randomly enrolled into the study. Eleven for control group were normally fed and provided with water drink, and 49 for experimental group were normally fed but provided with 1.2 g/L of thioacetamide instead of water to set up experimental liver fibrosis model [10]. During the course of 8^th^, 12^th^, and 16^th^ week, twelve experimental rabbits were picked out at random for examination, and the remaining experimental rabbits were handled at the 20^th^ week. Stage of liver fibrosis at different time intervals were presented in Table 1. Before the examination the animals were fasted for 6 h but had free access to water. Anesthesia was performed by intramuscular injection of 0.15 ml/kg of Sumianxin. The fur of hepatic region was shaved with 8% of sodium sulfide.

**Table 1:** Stage of liver fibrosis at different time intervals

	**F1**	**F2**	**F3**	**F4**
8w	11	1	0	0
12w	0	4	6	2
16w	0	0	9	3
20w	0	0	3	10

This study protocol complied with the guidelines for animal care of our institution and European community council directives. All procedures were reviewed and approved by the Animal Ethics Committee of the Fourth Military Medical University of the People’s Liberation Army of China (Xi’an, China).

### Doppler ultrasonography

Ultrasonography was performed using Sequoia 512 (Acuson Medical Ultrasonography Systems, USA) equipped with a linear array high frequency probe (8-13 MHz). The depth and instrument parameters remained unchanged during the course of examination. The liver was examined directly, and the superior mesenteric artery and splenic artery were detected using water-filled balloon method due to smaller diameters. Doppler sample volume was positioned in the center of the vessel and the sample width was selected to cover almost entire vessel diameter. Pulse repetition frequency was adjusted so as not to exceed the limit of the displayed maximum velocity. Care was taken to ensure that the angle of insonation was always smaller than 60 degree. Internal diameter of the vessels was measured manually after optimizing B mode images. The hemodynamic parameters such as the maximum, minimum and mean blood flow velocity (Vmax, Vmin, Vm), pulsatility index (PI) and resistance index (RI) were obtained. The hepatic circulate index (HCI) at different stages of liver fibrosis was calculated using the formula: HCI = PPV×PHA/SPPI (PPV: portal venous peak velocity; PHA: hepatic arterial peak velocity; SPPI: splenic arterial pulsatility index) [11,12]. All data were saved in machine-attached MO-CD for further analysis. The ultrasound operator was blinded with laboratory findings.

### Indocyanine green test

ICG (0.5 mg/kg of body weight) was injected via a peripheral vein. Serum was collected before and at 5, 10 and 15 minutes after the ICG injection to determine the ICG retention rate at 15 minutes (ICG R15). Each sample was diluted to one fourth of the concentration with saline, and the concentration of ICG in the specimens was analyzed by spectrophotometer at wave length of 805 nm. The calibration curve was made by the use of serum collected before the ICG injection in each rabbit and similarly manipulated. Retention value of less than 10 percentages at 15 min is considered to be within normal limits.

### Conventional liver function tests

The serum total bilirubin (T.B), albumin (ALB),alanine aminotransferase (ALT) and prothrombin time (PT) of the experimental animals were determined after ultrasound examination.

### Measurement of portal venous pressure

Portal venous pressure (PVP) was measured by a portal vein punctuation equipment.

### Histology examination

After examination, rabbits were sacrificed and liver specimens were fixed in formalin and embedded in paraffin. The slides stained by hematoxylin eosin (HE) and Masson three colors were evaluated by two independent expert pathologists. Liver fibrosis was evaluated according to the Xi’an meeting scoring system [13]. Fibrosis was staged as follows: no fibrosis (F0); F1- portal fibrosis without septa; F2- portal fibrosis and few septa; F3- numerous septa without cirrhosis; F4- cirrhosis. We found no significant difference in hemodynamics between stage F1 and F2 (data not shown), we thus combined these two stages as one. Thus, animals were divided into four groups: mild (F1-2), moderate (F3), advanced (F4) and control (F0) group according to fibrosis degree.

### Statistical analysis

The fibrosis stages were identified according to pathologic findings. Statistical analyses were carried out with SPSS 10.0 software (SPSS Inc., Chicago, IL, USA). All variables were expressed as the mean values ± standard deviation (mean±SD). A statistical analysis for continuous variable was performed using a student’s *t* test. For multiple values, ANOVA (analysis of variance) with LSD (least square design) was used. The agreement between the two measurements from two independent pathologists and between the two measurements from one pathologist at different time was performed. *P* value less than 0.05 was considered to represent statistically significant difference between tested data sets.

## Results

### Hepatic hemodynamics by ultrasound and correlation with the ICG R15

**Figure 1:**
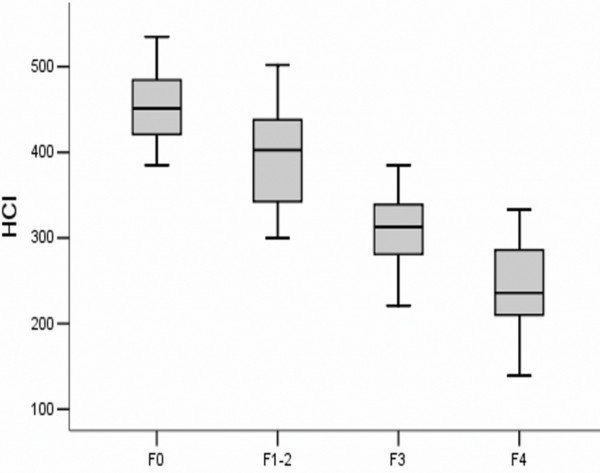
**HCI in different stages of fibrosis.** F0, without fibrosis; F1-2, mild fibrosis stage 1–2; F3, moderate fibrosis stage 3; and F4, advanced fibrosis stage 4.

The results of the ultrasound Doppler studies were summarized in Table 2. Portal venous velocity was not significantly different between groups. In contrast, HCI was significantly decreased in the advanced fibrotic stage (F4) compared to the mild (F1-2) and moderate fibrotic stages (F3), respectively (p<0.05). HCI was also significantly lower in F3 than that in F1-2 and F0 groups (p<0.05) (Figure 1).

**Table 2:** Hemodynamic data by Doppler ultrasonography (mean ± SD)

**Parameters**	**control (n=11)**	**mild (n=16)**	**moderate (n=18)**	**advanced (n=15)**
**Portal trunk**				
Diameter (mm)	3.68 ± 0.31	3.59 ± 0.30	3.63 ± 0.29	3.66 ± 0.25
Vm (cm/s)	14.3 ± 3.0	13.6 ± 3.5	13.4 ± 4.7	12.7 ± 3.2
FV (ml/min)	91 ± 8	82 ± 6	83 ± 6	80 ± 7
**Hepatic artery**				
Diameter (mm)	1.04 ± 0.23	1.12 ± 0.31	1.02 ± 0.19	1.05 ± 0.20
Vm (cm/s)	12.5 ± 4.8	13.2 ± 5.8	12.9 ± 5.1	13.7 ± 5.5
Vmax (cm/s)	26.3 ± 5.2	25.8 ± 5.5	26.3 ± 6.3	28.8 ± 10.8
**HCI**	452 ± 47	396 ± 56	308 ± 44	243 ± 55

Notes: Vm: Mean flow velocity; FV: Flow volume; Vmax: Peak systolic velocity; HCI: Hepatic circulation index.

**Figure 2:**
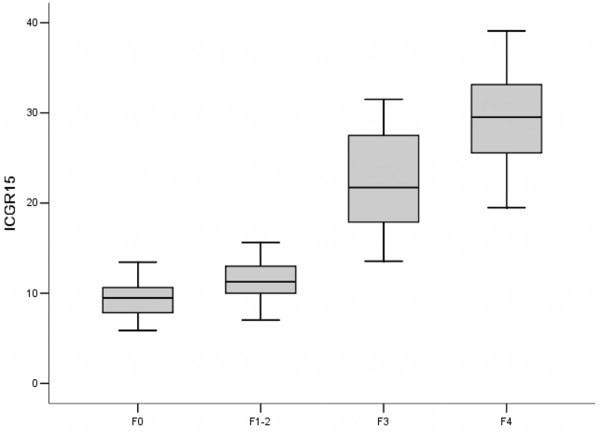
**ICG retention rate (percentage of total injected ICG dose) at 15 min (ICG 15) in different stages of fibrosis.** F0, without fibrosis; F1-2, mild fibrosis stage 1–2; F3, moderate fibrosis stage 3; and F4, advanced fibrosis stage 4.

**Figure 3:**
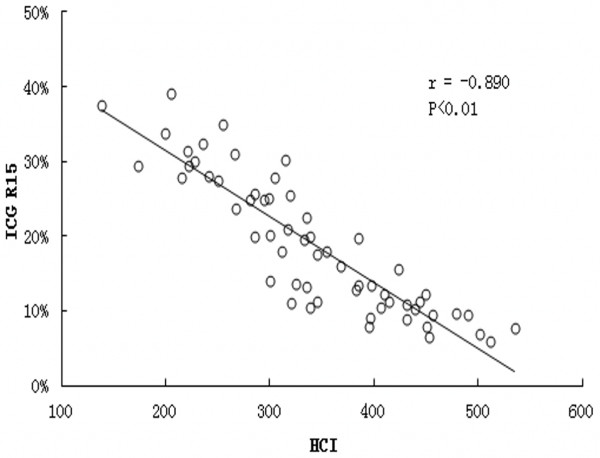
Correlation between HCI (Hepatic circulation index) and ICG retention rate (percentage of total injected ICG dose) at 15 min in different stages of fibrosis.

The ICG R15 in the fibrotic groups was significantly higher than that in the control group (0.209±0.086 vs. 0.093±0.023, p<0.01) (Figure 1). ICG R15 was higher in the advanced fibrotic stage (F4) than that in the mild (F1-2) or moderate (F3) fibrotic stage: 0.294±0.058 vs. 0.114±0.022 and 0.225±0.051, respectively (p<0.01) (Figure 2). A better inverse correlation was found between HCI and ICG R15 (*r* = −0.890, p <0.01) (Figure 3).

### Correlation of hepatic hemodynamics with PVP and liver function

**Figure 4:**
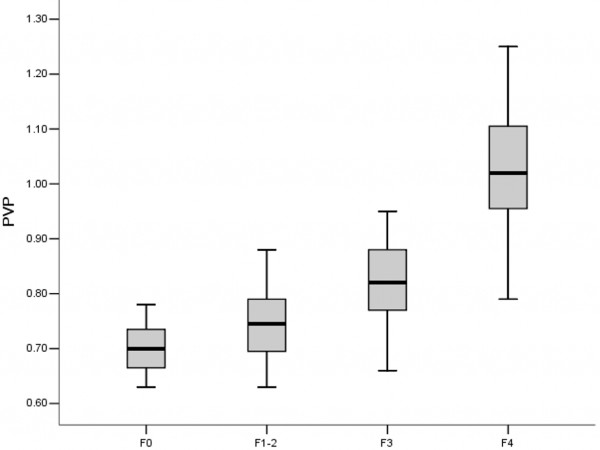
**PVP (Portal venous pressure) at the different stages of fibrosis in four sub-groups.** F0 (without fibrosis), F1-2 (mild fibrosis stage 1–2), F3 (moderate fibrosis stage 3) and F4 (advanced fibrosis stage 4).

**Figure 5:**
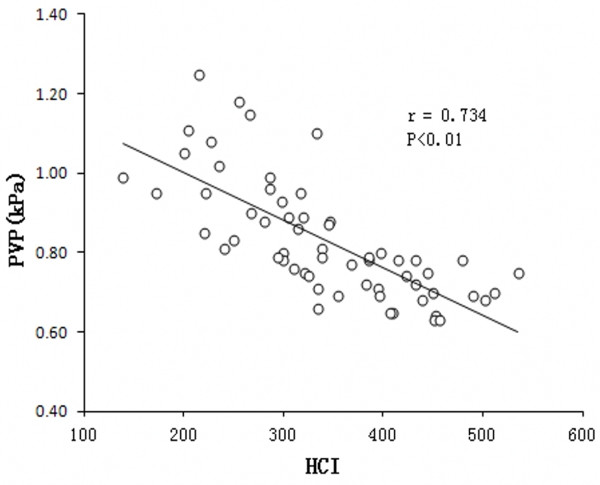
Correlation between PVP (Portal venous pressure) and HCI (Hepatic circulation index) at different stages of fibrosis.

**Figure 6:**
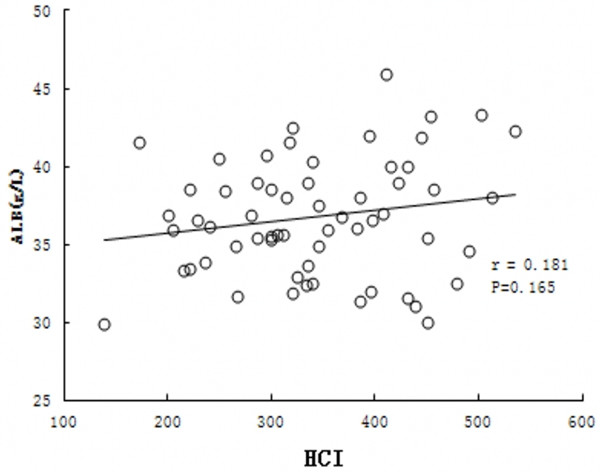
**Correlation between HCI (Hepatic circulation index) and liver function in fibrotic group.A**: serum albumin (ALB); **B**: serum total bilirubin (T.B); **C**: alanine aminotransferase (ALT).

Conventional liver function parameters of different degree of liver fibrosis were shown in Table 3. The difference of ALT between experimental groups and control group was significant (p<0.01). The results of PVP studies are depicted in Figure 4. The mean PVP in the fibrotic groups was significantly higher than that in the control group. PVP was higher in advanced fibrotic stage (F4) than those in mild (F1-2) or moderate (F3) fibrotic stage: 1.03±0.12 kPa vs. 0.74±0.06 kPa and 0.82±0.08 kPa, respectively (p<0.05) (Figure 4).With the development of fibrosis, PVP tended to increase and serum ALB decreased. A better correlation was found between PVP and HCI (*r* = 0.734, p<0.01) (Figure 5). On the other hand, there was no significant correlation between HCI and serum total bilirubin (*r*=0.109, p=0.407), serum albumin (p=0.165, *r* =0.181) and alanine aminotransferase (*r* =0.012, p=−0.437) (Figure 6 A-C).

**Table 3:** Conventional liver function tests (mean±SD)

	**control (n=11)**	**mild (n=16)**	**moderate (n=18)**	**advanced (n=15)**
ALT(U/L)	36±15	156±52	168±49	152±40
ALB(g/L)	37.3±5.7	37.2±3.7	36.8±2.6	35.6±3.4
T.B(umol/L)	8.6±2.4	8.5±3.3	9.2±3.6	8.2±4.7
PT(s)	11.5±1.3	10.1±1.5	11.9±0.9	10.7±1.8

ALT: Alanine aminotransferase; ALB: Albumin; T.B: Total bilirubin; PT, prothrombin.

### Inter and intra-observer variability

The agreement of staging the fibrosis was 94% for the two independent expert histopathologists and 100% for one of the expert histopathologists.

## Discussion

The present study demonstrated that Doppler parameters could identify different stages of hepatic fibrosis, and these Doppler parameters correlated closely with ICG retention rate (percentage of total injected ICG dose) at 15 min (ICG 15), which is considered to be one of the most valuable and reliable tests for assessing hepatic functional reserve and predicting post-hepatectomy liver failure in cirrhotic patients.

The assessment of hepatic functional reserve of patients with cirrhosis is critical for predicting prognosis, postoperative outcome in those who are candidates for nonhepatic surgery or liver resection for hepato-cellular carcinoma (HCC), or for determining the timing of liver transplantation in patients with advanced cirrhosis [14]. Quantitative assessment of hepatic function reserve before the occurrence of liver cirrhosis has been paid much attention in clinical practice in recent years. Though liver biopsy is the gold standard for diagnosing and staging liver fibrosis, its clinical application is limited because of its invasiveness. Exploring non-invasive methods for evaluating hepatic reserve function of liver fibrosis is of significance. Present study showed that ALT concentration was increased significantly after modeling, and remained at a high lever during the fibrosis stage. However, no significant difference in ALT was found between all experimental groups, indicating that the conventional hepatic function parameters are not sensitive in reflecting the hepatic reserve function changes at different stages of liver fibrosis. ICG retention rate at 15 min (ICG R15) is considered to be one of the most valuable and reliable tests for assessing hepatic functional reserve and predicting post-hepatectomy liver failure in cirrhotic patients. The present study also showed that with the development of liver damage, ICG R15 was increased while clearance ratio of indocyanine green decreased, and there was significant difference in ICG R15 among experimental groups. This might be related to the fact that the liver blood flow resistance was increased due to increased liver fiber tissue and hepatic sinusoidal endothelium capillarization, resulting in the decreased effective hepatic flow volume. These results suggest that ICGR15 could better reflect the severity of the liver fibrosis compared to the conventional hepatic function parameters.

Though ICG R15 is considered to be one of the most valuable and reliable tests for assessing hepatic functional reserve and predicting post-hepatectomy liver failure in cirrhotic patients, the measurement of ICG R15 is relatively complicated. The liver fibrosis is a pathological process of abnormal proliferation of hepatic fiber connective tissue in response to various kinds of liver-damaging factors, which is mainly characterized by the excessive deposition of extracellular matrix and hepatic sinusoidal endothelium capillarization. Advanced liver fibrosis results in the change of hepatic vascular structure and the increase of the hepatic vascular resistance, and even develops into portal hypertension. Actually in human, there is development of portal hypertension even in pre-cirrhotic stage. After development in portal hypertension, there are changes in the hepatic circulation. Thus, the hepatic hemodynamic parameters by US might change with the development of liver fibrosis. Although there was no significant difference in blood flow velocity of hepatic artery and portal venous system between the experimental group and the control group, HCI, a parameter that combines both the hepatic arterial and portal venous measurement, was decreased gradually with the development of fibrosis. Moreover, HCI has significant negative correlation with portal venous pressure and ICG R15, indicating that the liver and its peripheral organs were in high-resistance state since HCI could overall reflect the liver blood flow perfusion profile that correlated with hepatic reserve function [11]. These results indicate that HCImight be an alternative parameter of ICG R15 that could sensitively reflect the gradually-decreased hepatic reserve function during the course of different degree of liver fibrosis and portal hypertension.

## Conclusions

In conclusion, hepatic function reserve closely relates to the hepatic hemodynamics in the rabbit model of liver fibrosis. Doppler Ultrasound could be reliably used to assess the hepatic function reserve and hemodynamic changes at different stages of liver fibrosis.

## Competing interests

The authors declare that they have no competing interests.

## Authors’ contributions

YLY, LD and YYD was responsible for conception and participation in design, experimental work and collection of data, analysis and interpretation of results, drifting and substantial editing the manuscript. XL, RJY, SC were responsible for experimental work and collection of data, analysis and interpretation of results. LJY was responsible for interpretation of results and critically revising the manuscript. All authors read and approved the final manuscript.

## Pre-publication history

The pre-publication history for this paper can be accessed here:

http://www.biomedcentral.com/1471-230X/12/168/prepub
